# Clinical Pharmacology of Insulin Aspart Biosimilar GP40071: Pharmacokinetic/Pharmacodynamic Comparability in Hyperinsulinemic Euglycemic Clamp Procedure

**DOI:** 10.1002/cpdd.1084

**Published:** 2022-03-01

**Authors:** Roman V. Drai, Tatiana L. Karonova, Alexander Y. Mayorov, Igor E. Makarenko, Artem R. Dorotenko, Viktoria S. Kulesh, Vladislav V. Kovalik, Alena T. Andreeva

**Affiliations:** ^1^ R&D center GEROPHARM Saint‐Petersburg Russia; ^2^ Institute of Endocrinology Almazov National Medical Research Center Saint Petersburg Russia; ^3^ Endocrinology Research Centre Moscow Russia

**Keywords:** insulin aspart, biosimilar, pharmacokinetics, pharmacodynamics, hyperinsulinemic euglycemic clamp

## Abstract

Insulin aspart is a short‐acting insulin analogue that is used to control postprandial glycemia levels in diabetic patients. The aim of this clinical trial was to compare the pharmacokinetics and pharmacodynamics of GP40071 (GP‐Asp) and NovoRapid Penfill (Novo‐Asp) in a hyperinsulinemic euglycemic clamp (HEC). This trial was conducted as a part of a GP40071 biosimilar clinical development program. This was a phase I randomized, double‐blind, two‐period crossover study. Twenty‐six healthy male volunteers aged 18 to 45 years who met the inclusion criteria underwent the procedure of an HEC following a single subcutaneous injection of 0.3 IU/kg of either GP‐Asp or Novo‐Asp into the abdomen. After doses, plasma glucose levels were monitored every 5 minutes for 8 hours. The adjustment of the glucose infusion rate (GIR) was based on the blood glucose measurements. The GIR values were used to evaluate the PD profiles of the studied drugs. Regular blood sampling was performed during the study to obtain sufficient pharmacokinetic data. The 90% confidence intervals for the geometric mean ratios of the pharmacokinetic (AUC_ins.0‐t_, C_ins.max_) and pharmacodynamic (GIR_max_, AUC_GIR0‐t_) parameters of GP‐Asp were within the 80%–125% comparability limits. The safety profiles of the drugs were also comparable. Bioequivalence, similar PD, and safety of GP‐Asp and Novo‐Asp were demonstrated.

Insulin aspart (100 IU/mL) is a rapid‐acting insulin analogue. In insulin aspart, proline is replaced with aspartic acid in position 28 on the B‐chain of the regular human insulin (RHI). This modification allows insulin aspart hexamers to dissociate faster and thus it has a positive impact on the insulin pharmacodynamic profile in terms of shortening the onset of action.^1^

Postprandial reduction of glucose level is an important goal that provides better glycemic control in diabetes patients. Insulin aspart compared to RHI (with basal NPH insulin) found a slight but significant decrease in HbA1c and significantly lower postprandial blood glucose levels.^2^ This fact makes insulin aspart a socially important insulin analogue.

For over a decade insulin aspart has been commercially available as NovoRapid^®^ (Novo Nordisk).^3^ Recently two insulin aspart biosimilars were approved by the European Medicines Agency (EMA): Insulin Aspart Sanofi^®^ (Sanofi) and Kixelle^®^ (Biocon).

A biosimilar is a biological medicine highly similar to a referent medicine in terms of structure, biological activity, pharmacokinetic/pharmacodynamic (PK/PD) properties, efficacy, safety, and immunogenicity profile.^4^ The aim of insulin aspart biosimilar development is to reduce the cost and therefore make insulin products more affordable among diabetes patients. Cost reduction may be observed in both biosimilar insulin and in originator insulin due to economic competition between the two products. This phenomenon has an important socio‐economic impact.^5^

The process of insulin biosimilar development is a stepwise process that is strictly regulated. The nonclinical comparability program includes in vitro pharmacology studies and sometimes additional in vivo toxicological assessment. The clinical development program includes PK, PD, and safety studies as well as the risk management plan preparation. The main goal of clinical pharmacology assessment of biosimilar insulin analogues is to demonstrate the similarity of PK/PD profiles and, in consequence, the comparable efficacy of biosimilar and original products.

The hyperinsulinemic euglycemic clamp (HEC) study is the “gold standard” of PK/PD evaluation of the investigated biosimilar and reference insulin products. In the HEC approach the hypoglycemic effect of investigated insulins is antagonized by glucose infusion. Glucose infusion rate (GIR) is adapted according to glycemia and the GIR values are recorded to fit the GIR–time curve, that is, the PD profile. To assess the PK profile of the insulin products, venous blood sampling was carried out during the whole HEC procedure.^6^

Biosimilar of rapid‐acting insulin aspart GP40071 is being developed. Preliminary head‐to‐head studies have been performed to demonstrate comparability of biosimilar and reference drugs by sensitive analytical methods and preclinical in vitro paradigms.

The aim of this study was to assess the PK/PD comparability of GP‐Asp and Novo‐Asp in healthy male volunteers using the HEC procedure.

## Materials and Methods

### Study Design

This was a phase 1, randomized, double‐blind, two‐center, two‐treatment, single‐dose, two‐period, crossover, 8‐hour HEC study in healthy subjects. The study was conducted simultaneously in 2018 at two clinical sites (Almazov National Medical Research Center, Saint Petersburg, Russia, and Endocrinology Research Centre, Moscow). All trial procedures were performed in accordance with Good Clinical Practice guidelines and ethical principles for medical research involving human subjects established by the Declaration of Helsinki. All study participants provided written informed consent before entering the study. The trial protocol was reviewed and approved by the Ministry of Health of Russian Federation (Clinical trial authorization No 502, September 27, 2018) and by the independent ethics committee at each research center. The study is registered at ClinicalTrials.gov with ID: NCT04184466.

After screening (up to 14 days), individuals enrolled in the study were randomly assigned to one of two groups. The first group received the test treatment (T) in the first period and the reference treatment (R) in the second period. The second group, vice versa, received R in the first period and T in the second period. The washout period between two treatment visits lasted 7–14 days. A final follow‐up visit was arranged 7–14 days after the second treatment was administered.

### Study Population

Eligible subjects included healthy Caucasian men aged 18–45 years with a body mass index (BMI) between 18.5 and 30.0 kg/m^2^, both inclusive, and a body weight between 55 and 100 kg, both inclusive.

Individuals with any acute or chronic diseases, known allergies, as well as laboratory or vital sign abnormalities were excluded at screening. Subjects were also excluded if they had an elevated fasting venous blood glucose>>109.8 mg/100 mL, glycemia level ≥140.4 mg/100 mL 2 hours after the glucose load, and HbA1C > 6% at screening. Other exclusion criteria comprised smoking, high alcohol consumption, taking any medicines in the last 2 weeks, following any diet, history of recent blood loss, known episodes of hypoglycemia, and family history of diabetes. The full list of exclusion criteria is available at ClinicalTrials.gov.

### Treatments

The test drug, GP40071 (GP‐Asp), as a solution for injection (100 IU/mL) was manufactured by GEROPHARM, Russia. NovoRapid^®^ Penfill^®^ (Novo‐Asp), as a solution for injection (100 IU/mL), by NovoNordisk, Denmark was used as a reference drug. Both drugs were administered subcutaneously into the abdomen using a BD Micro‐Fine 0.5‐mL insulin syringe with an 8‐mm needle. In this study, the dose of insulin aspart was 0.3 IU/kg for each formulation.

### Sample Size Estimation

AUC_ins.0‐t_ and C_ins.max_ variability were analyzed based on similar trials. According to previous studies,^6^ an intrasubject variability of 19% was assumed as the most variable parameter for C_ins.max_. Considering this variability, 26 volunteers (considering possible dropouts) were randomized to provide at least 90% power to show the 90% confidence interval (CI) of the ratio of means for C_ins.max_ and AUC_ins.0‐t_ between the two drugs to be within the 0.8 and 1.25 bioequivalence limits.

### Hyperinsulinemic Euglycemic Clamp Procedure

The PD effect of insulin aspart was evaluated using the manual euglycemic clamp technique, as described previously^7^ with adaptations described below.

*Before the clamp procedure*. Subjects were hospitalized approximately 12 hours before each drug administration. After the baseline physical examination, vital signs measurements, alcohol breath test, and urine drug testing were performed to confirm compliance with the study restrictions. All participants were given a standardized light meal in the evening before the clamp. Subjects fasted for at least 10 hours before the dosing and remained fasted until the end of the clamp.

*During the clamp procedure*. The next morning HEC was performed. Subjects remained in a supine position for the entire procedure. The time of dosing of the investigated insulins was defined as a zero point. Plasma glucose levels were measured to confirm euglycemia state 60 and 30 minutes prior to the dosing. The target value for blood glucose concentrations during the clamp procedure was defined as 90 mg/100 mL and the acceptable glycemic range in this study was set as 80–100 mg/100 mL. After subcutaneous injection of the investigated insulin, intravenous 20% glucose infusion was started once the baseline plasma glucose level decreased by more than 5 mg/100 mL. The glucose infusion rate (GIR) was controlled and adjusted manually using an infusion pump (Infusomat fmS, B. Braun Melsungen AG, Germany) to maintain plasma glucose concentrations of 80–100 mg/100 mL. HEC lasted 8 hours, and during the entire procedure glycemia was measured using a glucose analyzer (StatStrip, Nova Biomedical, USA) and GIR was adjusted accordingly every 5 minutes. The GIR was documented throughout the procedure and was used to reflect the activity of insulin. Glucose infusion could be discontinued earlier if GIR equaled 0 mg/kg/min at three consecutive measurements 6 hours or more after dosing. Blood samples for PK assessment were obtained for 8 hours postdose.

HEC quality was assessed by calculating mean plasma glucose levels and coefficient of variation (CV), reflecting HEC precision.

### Data Analyses

*Pharmacokinetics*. During each HEC 21 samples (each 9 mL of venous blood) were collected from all participants at regular time intervals to assess the concentrations of insulin aspart and C‐peptide: 60, 30, 0 minutes predose and 10, 20, 30, 40, 50, 60, 75, 90, 105, 120, 135, 150, 165, 240, 300, 360, 420, 480 minutes postdose. To obtain plasma samples for the bioanalytical part the blood samples were centrifuged at 3000 rpm for 15 minutes, then the supernatant was transferred into clean tubes. Insulin determination in plasma was performed by a validated enzyme immunoassay (EIA) method using a Personal Lab (Adaltis S.r.l., Italy) analyzer. A Mercodia Iso‐Insulin ELISA kit (Mercodia, Sweden) was used to measure total endogenous human insulin and insulin aspart concentrations and then a Mercodia Insulin ELISA kit (Mercodia) was used to quantify specifically the endogenous human insulin concentration. This bioanalytical technique was based on a previously described approach.^8^ The validated lower (LLOQ) and upper (ULOQ) levels of quantification for total insulin assay were 10.00 and 300.00 μIU/mL, and for human insulin assay they were 10.00 and 150.00 μIU/mL. The concentrations of insulin aspart were determined as the difference between the values obtained during the analysis of samples by both assays above (double measurement technique). C‐peptide concentration in plasma was also measured using a DRG C‐peptide ELISA kit (DRG Instruments, Germany)^9^ using a Personal Lab (Adaltis S.r.l.) analyzer with LLOQ and ULOQ as 0.20 and 16.00 ng/mL, respectively. All procedures were performed according to the available manufacturers’ instructions. In these assays all samples were analyzed in duplicate and concentrations below the low level of quantitation were treated as zero. Additional data obtained during the validation process of both bioanalytic methods are presented in Table 1.

**Table 1:** Validation Parameters of Bioanalytical Methods

Parameters	Insulin Aspart Detection Method	C‐Peptide Detection Method
Standard calibration curve (LLOQ‐ULOQ)	10–300 μIU/mL	0.2–16.0 ng/mL
Accuracy (within‐run), % of nominal	LLOQ 7.62% ULOQ 5.25% L, M, H conc. ≤6.22%	≤14.95%
Accuracy (between runs), % of nominal	L, M, H conc. ≤4.91%	≤6.17%
Precision (within‐run), CV	LLOQ 8.00% ULOQ 1.11% L, M, H conc. ≤4.43%	LLOQ 5.00% ULOQ 0.51% L, M, H conc. ≤7.24%
Precision (between runs), CV	≤6.95%	≤9.92%

conc., concentration; СV, coefficient of variation; H, high concentration level; L, low concentration level; LLOQ, lower limit of quantification; M, mid concentration level; ULOQ, upper limit of quantification.

PK endpoints included the area under the concentration versus time curve from time zero to the end of clamp period at time t (AUC_ins.0‐t_), maximum insulin concentration detected (C_ins.max_), time to peak plasma insulin concentration (t_max_), insulin half‐life (t_1/2_), and areas under the concentration versus time curve from time zero to 1, 3, and 5 hours after insulin injection (AUC_ins.0‐1_, AUC_ins.0‐3_, and AUC_ins.0‐5_, respectively) as well as the area under the concentration versus time curve extrapolated to infinity (AUC_ins.0‐∞_).

*Pharmacodynamics*. As described above, blood glucose values determined every 5 minutes during HEC were used to measure and manually adjust GIR. Based on GIR values, the following PD endpoints were estimated: area under the GIR versus time curve from time zero to the end of clamp period at time t (AUC_GIR0‐t_), maximum GIR (GIR_max_), time to maximum GIR (tGIR_max_), time from insulin injection to the start of glucose infusion (tGIR_lag_), and areas under the GIR versus time curve from time zero to 1, 3, and 5 hours after insulin injection (AUC_GIR0‐1_, AUC_GIR0‐3_, and AUC_GIR0‐5_, respectively).

*Safety evaluation*. The safety and tolerability of both studied drugs were also assessed. Throughout the trial, adverse events (AEs) were monitored and all participants underwent physical examination, vital signs assessment, electrocardiogram (ECG) recording, and laboratory blood and urine testing.

### Statistical Analyses

Calculation of descriptive statistics parameters, bioequivalence, and PD comparability assessment were performed using R software version 3.5.0.

To assess bioequivalence, geometric means (GMs) for GP‐Asp and Novo‐Asp were compared and 90%CI was estimated for the ratios of GMs. The log‐transformed AUC_ins.0‐t_ and C_ins.max_ were assessed using ANOVA. The terms included in ANOVA were treatment, subject, sequence, and period. Mean square error (MSE) obtained from ANOVA was used to estimate the ratio of GM and 90%CI. Equivalent bioavailability was concluded if the 90%CI for the ratio of GP‐Asp to Novo‐Asp for AUC_ins.0‐t_ and C_ins.max_ was completely within the limits of the 0.80–1.25 interval. The similarity of the key PD parameters (GIR_max_, AUC_GIR0‐t_) was analyzed as described for bioequivalence.

## Results

### Baseline Characteristics of Subjects

A total of 26 healthy male volunteers were randomized for this study. All subjects completed the study and were included in the analysis of pharmacokinetics and pharmacodynamics . Baseline participant characteristics are represented in Table 2. Subjects had similar baseline characteristics across the sequence groups.

**Table 2:** Baseline Characteristics of Subjected Volunteers

Characteristics	Subjects (N = 26), Mean ± SD/% of N
Age, years	29.54 ± 4.56
Gender (males)	26 (100.0%)
Ethnicity (Caucasian)	26 (100.0%)
Body weight, kg	80.71 ± 8.97
Height, cm	180.54 ± 3.8
BMI, kg/m^2^	24.75 ± 2.48
Smokers Yes No Previously	0 (0.0%) 26 (100.0%) 0 (0.0%)
HbA1c, %	5.18 ± 0.3
2 hours plasma glucose level after OGTT (mg/100 mL)	86.4 ± 19.6

BMI, body mass index; HbA1c, glycated hemoglobin; N, number of randomized subjects; OGTT, oral glucose tolerance test; SD, standard deviation.

### Pharmacokinetics

**Figure 1:**
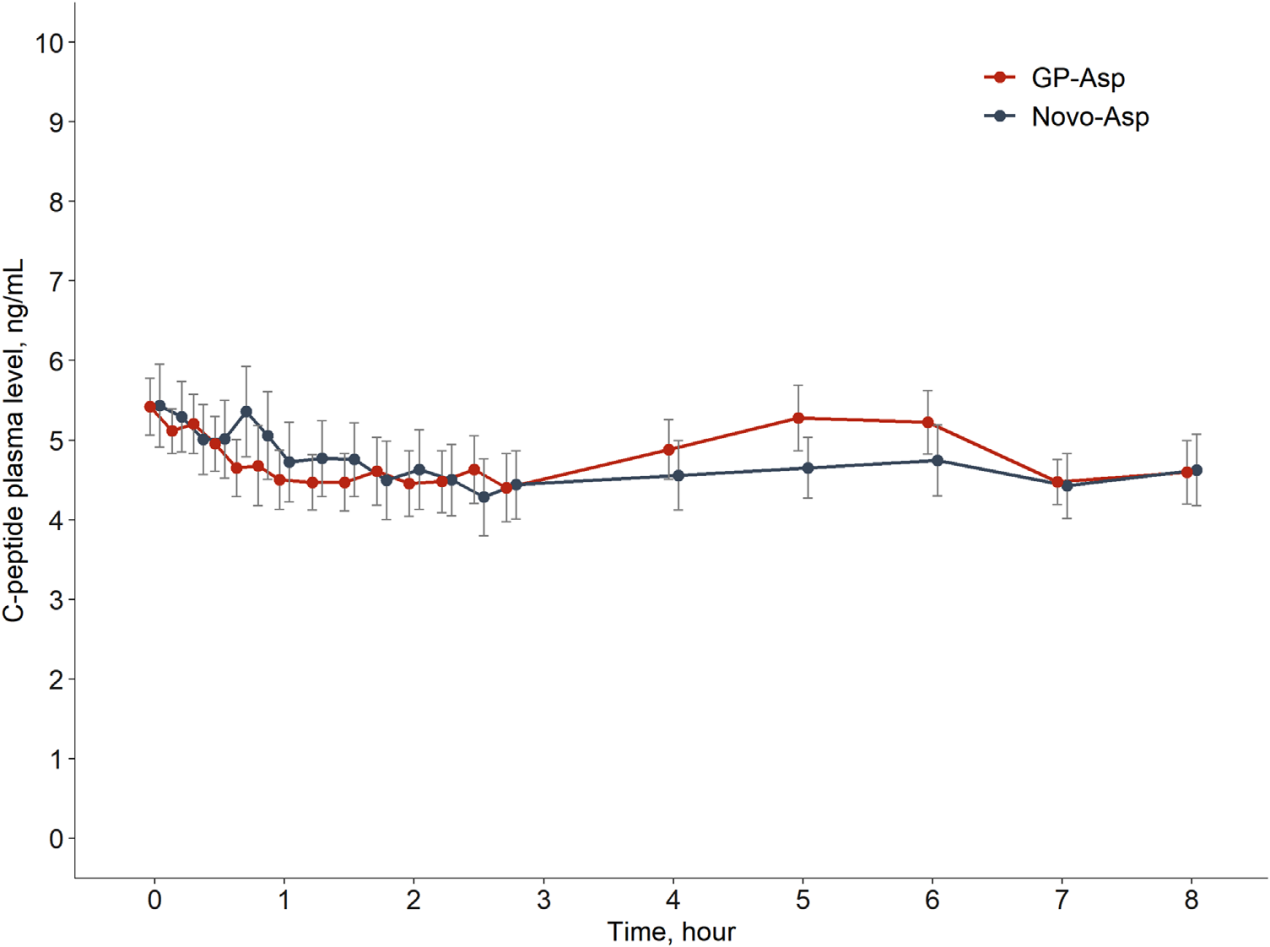
C‐peptide plasma levels during both HEC procedures after GP‐Asp and Novo‐Asp administration (mean ± SE, N = 26). All values below the low level of quantitation have been entered as zero and included in the calculation of means.

The mean C‐peptide plasma levels were similar after GP‐Asp and Novo‐Asp injection (Figure 1). During HEC no fluctuations in C‐peptide concentration were registered, indicating that no peak endogenous insulin production occurred.

**Figure 2:**
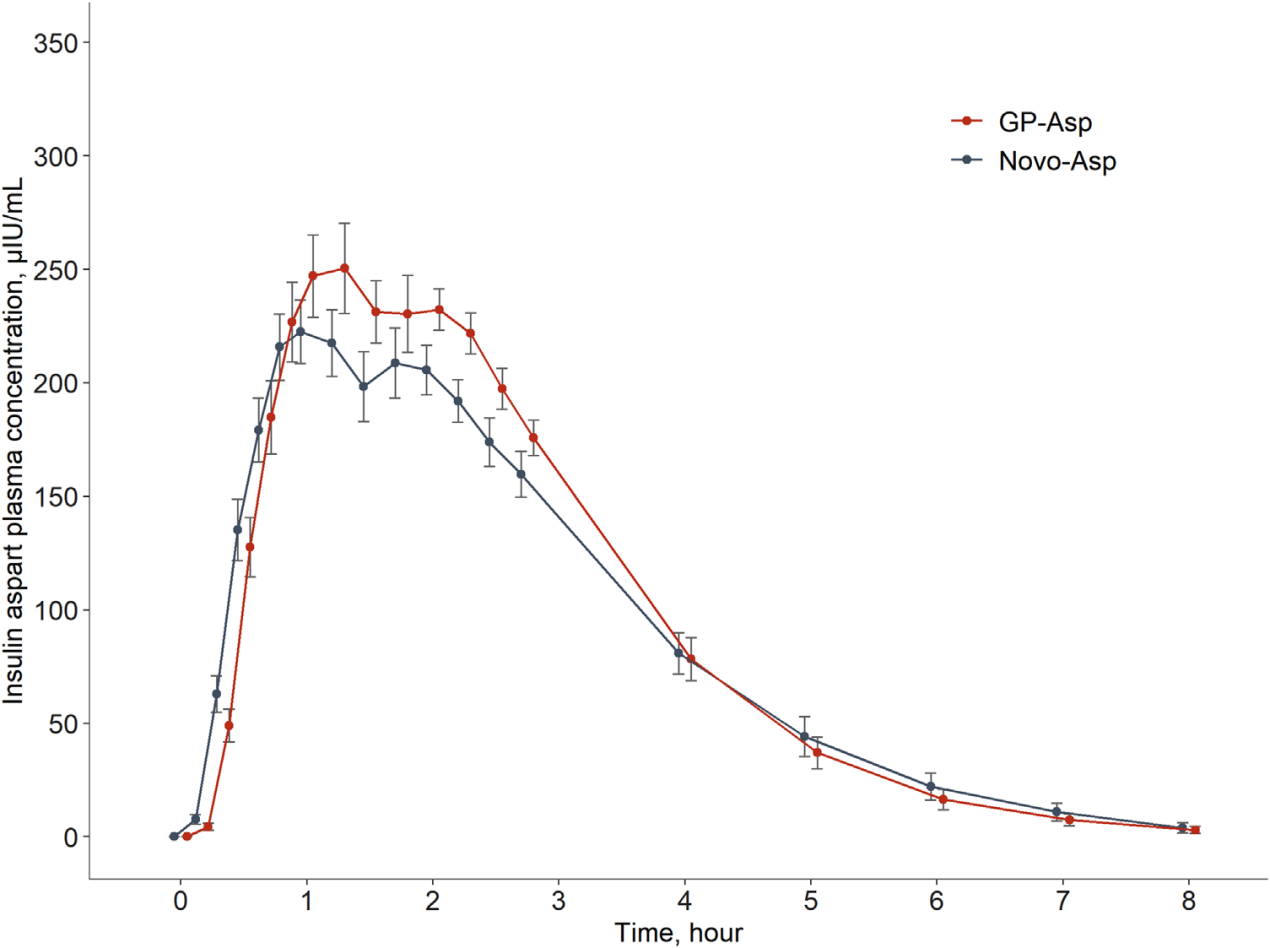
Insulin aspart plasma concentrations during both HEC procedures after GP‐Asp and Novo‐Asp administration (mean ± SE, N = 26). All values below the low level of quantitation have been entered as zero and included in the calculation of means.

The PK profiles of GP‐Asp and Novo‐Asp had similar shapes, as depicted in Figure 2. The similarity was also confirmed by the AUC_ins.0‐t_ and C_ins.max_ GM ratios contained within the 0.80–1.25 interval. For AUC_ins.0‐t_ the T/R ratio (90%CI) was 1.06 (102.12‐110.24) and for C_ins.max_ the T/R ratio (90%CI) was 1.17 (110.51‐124.02). Thus, equivalent exposure of GP‐Asp and Novo‐Asp was demonstrated. Descriptive statistics for each of the PK endpoints as well as GM ratios are shown in Table 3.

**Table 3:** Pharmacokinetic (PK) and Pharmacodynamic (PD) Endpoints (Healthy Volunteers, N = 26)

PK/PD	Endpoint	GP‐Aspmean (SD)	Novo‐Aspmean (SD)	GP‐Asp/Novo‐AspGmean ratio (90%CI)
PK	AUC_ins.0‐t_, μLU/mL × h	750.86 (163.48)	716.37 (199.60)	1.06 (102.12%–110.24%)
PK	C_ins.max_, μLU/ml/mL	286.95 (86.53)	245.58 (72.70)	1.17 (110.51%–124.02%)
PK	AUC_ins.0‐∞_, μLU/ml/mL × h	776.90 (166.33)	745.70 (209.72)	–
PK	t_max_, h	1.51 (0.53)	1.37 (0.49)	–
PK	t_1/2_, h	0.88 (0.31)	0.93 (0.36)	–
PD	AUC_GIR0‐t_, mg/kg × 60 min	43.48 (12.43)	42.81 (13.28)	1.02 (91.09%–113.84%)
PD	GIR_max_, mg/kg/min	11.21 (3.43)	10.65 (3.3)	1.05 (94.35%–117.53%)
PD	t_GIRmax_	2.75 (0.94)	2.82 (1.02)	–
PD	t_GIRlag_	0.48 (0.21)	0.44 (0.18)	–

AUC, area under the curve; C_ins.max_, maximum plasma concentration of insulin aspart; CI, confidence interval; Gmean, geometric mean; GIR_max_, maximal glucose infusion rate; t_max_, time to reach C_ins.max_; t_1/2_, half‐life; mean, arithmetic mean; t_GIRmax_, time to reach GIR_max_; t_GIRlag_, onset of action; SD, standard deviation.

### Pharmacodynamics

**Figure 3:**
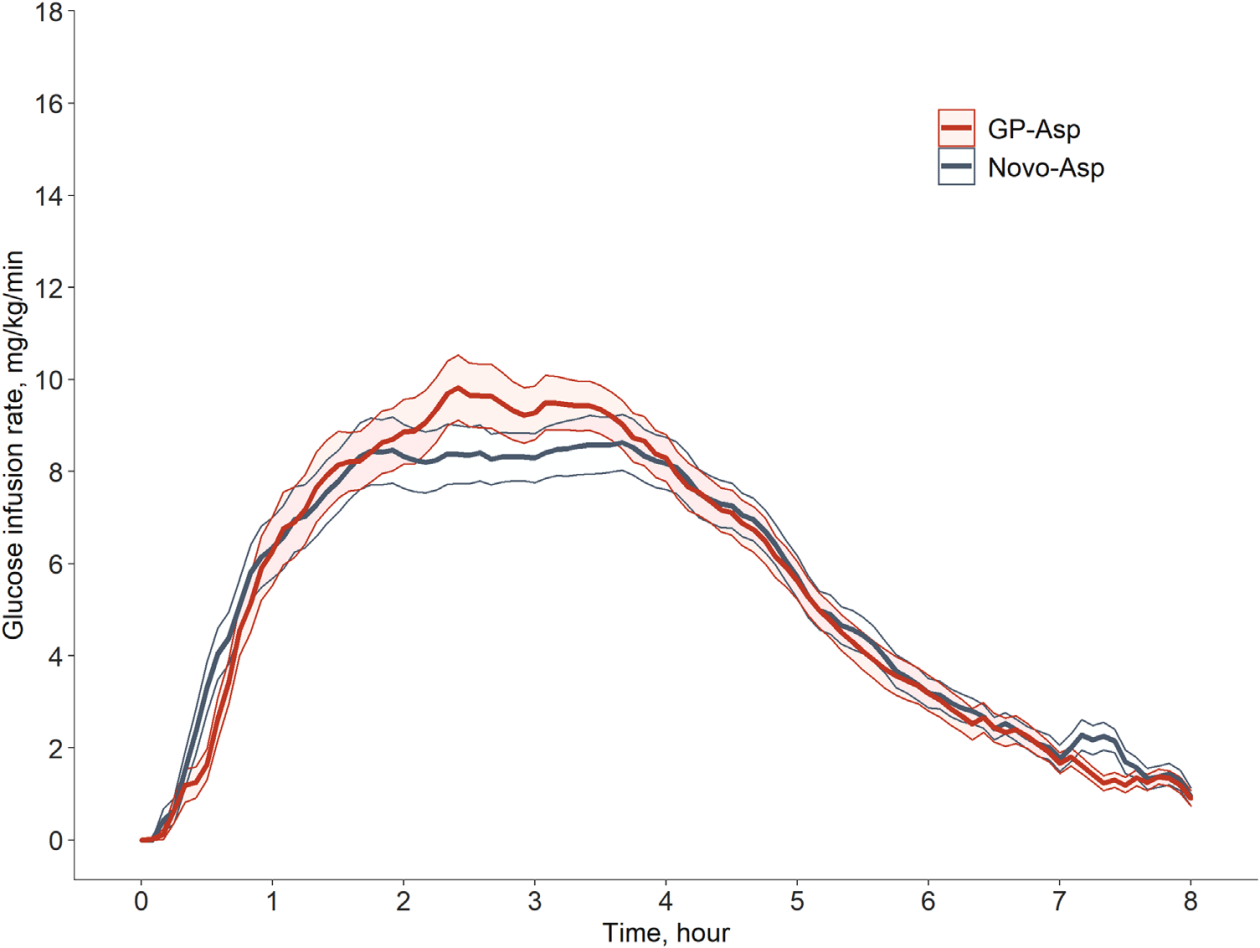
Glucose infusion rate (GIR) during both HEC procedures after GP‐Asp and Novo‐Asp administration (mean ± SE, N = 26). Heavy lines represent means, shaded areas of each color are mean ± SE range, upper and lower thin lines of each color are mean + SE and mean − SE values, respectively.

The mean GIR profiles were similar after GP‐Asp and Novo‐Asp 0.3 IU/kg injection (Figure 3), suggesting a comparable blood glucose‐lowering effect. The 90%CI for GM ratios for the main PD parameters (AUC_GIR0‐t_ and GIR_max_) fell within the limits of 0.80–1.25. For AUC_GIR0‐t_ the T/R ratio (90%CI) was 1.02 (0.91‐1.14) and for GIR_max_ the T/R ratio (90%CI) was 1.05 (0.94‐1.18). Time to onset of insulin action and time to maximum GIR were also similar between formulations. Descriptive statistics for the PD endpoints are represented in Table 3.

### HEC Quality

**Figure 4:**
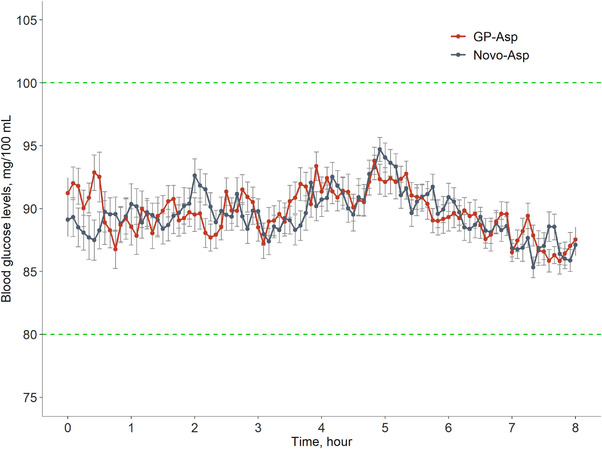
Blood glucose levels during both HEC procedures after GP‐Asp and Novo‐Asp administration (mean ± SE, N = 26). Green dotted lines represent the acceptable glycemic range (80–100 mg/100 mL).

The mean plasma glucose level for both GP‐Asp and Novo‐Asp during HEC was 90.27 mg/100 mL and coefficient of variation (CV%) of blood glucose measurements were 6.25% for GP‐Asp and 6.30% for Novo‐Asp. Plasma glucose levels during the HEC procedure are presented in Figure 4.

### Safety and Tolerability

All subjects completed 8 hours of each HEC procedure with no premature terminations. Single doses of both drugs were well tolerated. Four AEs, two in the GP‐Asp group and two in the Novo‐Asp group, were reported. One case of hyperbilirubinemia and one case of phlebitis were observed in each treatment group. All AEs were transitory and mild in intensity, and were most probably related to the HEC procedure.

## Discussion

In this single‐dose two‐period crossover HEC study trial carried out in healthy volunteers, it was demonstrated that a potential biosimilar drug GP‐Asp is bioequivalent to Novo‐Asp. The PD profiles of GP‐Asp and Novo‐Asp are also comparable. Single doses of both formulations were well tolerated and most AEs were related to HEC procedures. Clamp quality was confirmed by the evaluation of glycemia data obtained during the study periods.

According to the previous insulin aspart HEC studies, the 0.3 IU/kg dose was chosen to detect potential differences in the PK profiles of the two insulins. AUC_ins.0‐t_ and C_ins.max_ were used to assess the bioequivalence of these formulations. The obtained PD data (AUC_GIR0‐t_, GIR_max_) for the investigated insulins support the bioequivalence results.

Healthy volunteers enrolled in the study represent a homogeneous and insulin‐sensitive population, which allows the drug‐derived effects to be better assessed. Enrolling healthy volunteers as the subject population in bioequivalence studies allows the variability not related to differences between products to be reduced. This approach is also applicable for the comparability of rapid‐acting insulin preparations in PK/PD studies.^7^ Male volunteers were subjected in this study to exclude insulin sensitivity variation in the female population, which could influence the study results.

In this study, endogenous insulin secretion was suppressed by clamping blood glucose during the HEC procedure. C‐peptide was monitored in parallel to insulin concentrations to evaluate the levels of endogenous insulin release. A double measurement technique in the bioanalytical part of the study was performed to differentiate endogenous human insulin from administered insulin analogue. This approach provides the possibility of assessing the pharmacokinetics of insulin aspart and excluding endogenous insulin production affecting PK evaluation.

## Conclusions

In conclusion, bioequivalence and similar glucose‐lowering activity were confirmed for the test and reference products in the clamp study with subcutaneous administration of 0.3 IU/kg of test product and reference insulin aspart preparations in healthy subjects.

## Conflicts of Interest

This study was funded by GEROPHARM, Russia. The authors´ team includes employees of GEROPHARM.

## Author Contributions

RD and IM designed and directed the project. AM, TK, and AA performed the clinical part as investigators. VK analyzed the data. All authors discussed the results and contributed to the final manuscript. AD and VK wrote the manuscript.
